# Sports facilities as substitutes for exercise-induced well-being: Evidence from four-wave CFPS panel data

**DOI:** 10.1371/journal.pone.0359511

**Published:** 2026-09-30

**Authors:** Yang Wang, Liman Wang

**Affiliations:** 1 University of Jinan, Jinan, Shandong, China; 2 Shandong Urban Construction Vocational College, Jinan, Shandong, China; Dongshin University, KOREA, REPUBLIC OF

## Abstract

**Background:**

The relationship between sports participation and psychological well-being is well-documented, yet empirical evidence regarding the moderating role of community sports facilities remains inconclusive. This study tests whether facilities amplify or attenuate the well-being returns to exercise, using four-wave panel data from China (CFPS 2014, 2018, 2020, 2022).

**Methods:**

Linear regression models with individual fixed effects were constructed using China Family Panel Studies data (CFPS 2014, 2018, 2020, and 2022; the 2016 wave was excluded because its happiness item reached only 2.5% of adults). The analytical sample comprised 71,326 observations from 23,818 individuals. Self-rated happiness (0−10 scale) was regressed on binary exercise participation, community sports facility count (0−5+), and their interaction, controlling for age and log household income. Robustness was tested via instrumental variable estimation (community mean exercise rate) and propensity score matching.

**Results:**

Exercise positively predicted happiness (β = +0.074, p < 0.001) in the baseline fixed-effects model. The exercise × facility interaction was directionally consistent with the substitution hypothesis but not statistically significant (β = −0.022, p = 0.075). Marginal exercise effects declined from +0.095 in zero-facility communities (p < 0.001) to non-significance where three or more facility types were available. Instrumental variable estimation results (first-stage F = 3231.25; second-stage β = −0.158, p = 0.117). The IV estimate did not reach statistical significance, providing limited evidence on treatment effect heterogeneity. Four-wave balanced panel estimates confirmed the baseline finding (β = +0.074, p < 0.01). CES-D mediation accounted for only approximately 4.6% of the total effect.

**Conclusions:**

Exercise benefits subjective well-being most where community sports facilities are least available—a directionally consistent substitution pattern. Facility investment may yield greater marginal returns when targeted at underserved communities.

## Introduction

The psychological well-being of the Chinese population and their relatively low levels of sports participation have become pressing concerns in public health research [1,2]. In China, approximately 16.6% of the population experiences some form of mental disorder over their lifetime [1], and only about 33.9% of adults regularly participate in physical exercise [2].

A substantial body of experimental and epidemiological research has established that regular structured physical exercise improves mental health outcomes [3]. Meta-analytic evidence from randomized controlled trials demonstrates moderate-to-large effect sizes of exercise interventions on depression (SMD = −0.66 to −1.11) [3]. Several physiological mechanisms have been proposed: exercise modulates endorphin release [4], and may also reduce systemic inflammation, upregulate brain-derived neurotrophic factor (BDNF), and regulate hypothalamic-pituitary-adrenal (HPA) axis activity through broader neurobiological pathways [5].

From a sociological perspective, sports participation extends beyond individual-level physiological effects. Exercise provides structured opportunities for social interaction, identity formation, and community belonging—collectively described as the “social cure” mechanism [6]. Cross-sectional evidence suggests that physical activity has significant effects on depression and anxiety in non-clinical populations [7].

However, critical gaps persist in the literature. First, most prior studies rely on cross-sectional data, which leaves causal direction ambiguous: do happier people select into exercise, or does exercise causally improve well-being? Individual fixed-effects panel models address time-invariant unobserved confounders (e.g., personality traits, genetic predispositions), yet relatively few studies in the Chinese context have employed multi-wave panel designs [8].

Second, and more centrally, the moderating role of community-level sports resources—particularly the availability of sports facilities—remains theoretically underspecified. A recent study by Li et al. using a single cross-section of the China General Social Survey (CGSS) found that sports facilities moderate the relationship between exercise and psychological well-being [8]. But the theoretical mechanism underlying this moderation is ambiguous. Two competing hypotheses exist:

H1 (Complementarity Hypothesis). Community sports facilities amplify the well-being benefits of exercise. Well-equipped communities provide safer, more diverse, and more socially engaging exercise environments, magnifying the psychological returns. Under this hypothesis, the exercise x facility interaction term should be positive.

H2 (Substitution Hypothesis). Community sports facilities substitute for individual exercise effort in generating well-being. In facility-scarce communities, exercise requires greater personal initiative, effort, and mastery—qualities that independently contribute to psychological well-being through self-efficacy and competence mechanisms [9]. When facilities are abundant, these self-generated psychological benefits are partially offset by the convenience-driven, lower-effort nature of facilitated exercise. Under this hypothesis, the interaction term should be negative.

Third, the mechanism linking exercise to well-being is often assumed to operate through depression reduction, but this is rarely tested directly. If the mediation is weak (as preliminary evidence suggests), then interventions targeting depression alone may miss the primary well-being mechanism [10].

This study addresses these three gaps using four waves of the China Family Panel Studies (CFPS 2014, 2018, 2020, 2022). We make four contributions: (i) panel fixed-effects identification of the exercise-well-being relationship in the Chinese population; (ii) a formal empirical test of the competing complement-vs.-substitute hypotheses for facility moderation; (iii) CES-D mediation decomposition to quantify the depressive-symptom pathway; and (iv) instrumental variable estimation exploring instrumented exercise effect heterogeneity between voluntary and community-normed exercise.

## Materials and methods

### Data source

We used data from the China Family Panel Studies (CFPS), a nationally representative longitudinal survey conducted by the Institute of Social Science Survey at Peking University since 2010 [11]. The CFPS employs a multi-stage probability-proportional-to-size (PPS) sampling design covering 25 provinces/municipalities/autonomous regions, representing approximately 95% of the Chinese population. The survey collects individual-, household-, and community-level information across domains including demographics, socioeconomic status, health behaviors, and subjective well-being. The CFPS data for this study were accessed on 24 May 2026 via the Peking University Open Research Data platform (https://opendata.pku.edu.cn).

We utilized all four available waves of the CFPS: 2014, 2018, 2020, and 2022. Although the 2014 wave employed different response formats for the happiness item (qm2011) and the exercise frequency variable (qp701), these were harmonized with later waves: happiness responses were recoded to the 0–10 scale, and exercise participation was binarized (≥1 time/week = 1) using the available frequency categories. The eight-item CES-D depressive symptom scale was not fielded in 2014; therefore, CES-D mediation analyses use the 2018–2022 subsample only. The 2014 community questionnaire was also retained for its sports facility data (ca201a12), which provide time-invariant contextual measures linked to all waves through household identifiers. The 2016 wave was excluded because the happiness question was administered to only a small subsample (N = 844; 2.5% of adult respondents), precluding its use in panel analysis.

The derivation of each analytical subsample is summarized in Table 1. The raw CFPS panel (2014–2022) comprises 132,653 person-year observations from 52,373 individuals. We first restricted the sample to individuals appearing in at least two survey waves (116,339 observations, 38,680 individuals) to enable individual fixed-effects estimation, then matched these records to the 2014 community facility roster (81,869 observations, 26,637 individuals). After excluding observations with missing dependent or control variables, the complete-case analytical sample comprised 73,477 observations from 25,969 individuals (full sample). The FE estimation sample requiring ≥2 waves within complete cases comprised 71,326 observations from 23,818 individuals.

**Table 1:** Sample construction flow for analytical subsamples.

Stage	Criterion	Observations retained	Individuals retained	Excluded
Raw CFPS panel	All four waves (2014, 2018, 2020, 2022); 2016 excluded (happiness administered to 2.5% of adults only)	132,653	52,373	---
Panel participation	≥2 survey waves	116,339	38,680	−16,314
Community match	2014 facility data	81,869	26,637	−34,470
Complete cases	Key vars non-missing	73,477	25,969	−8,392
FE estimation sample	≥2 waves (for FE)	71,326	23,818	2,151
CES-D subsample	2018-2022 only (CES-D not fielded in 2014), ≥ 2 waves	46,306	18,155	---
Interaction subsample	With facility data (for FE)	71,326	23,818	---
Balanced panel	Present in all 4 waves (2014, 2018, 2020, 2022)	31,572	7,893	---
IV/PSM subsample	Full CFPS, community ID available	71,313	---	---

### Variables

Dependent variable. Subjective well-being was measured using a single-item happiness question: “How happy do you feel?” (CFPS variable qm2011; available in all four waves 2014–2022). Responses ranged from 0 (“very unhappy”) to 10 (“very happy”), with higher scores indicating greater well-being. This single-item measure has demonstrated acceptable psychometric properties in large-scale population surveys and correlates strongly with multi-item well-being scales (r > 0.70) [12].

Primary independent variable. Exercise participation was operationalized as a binary indicator coded 1 if the respondent reported physical exercise at least once per week. In CFPS 2018, the question was “How frequently do you participate in physical exercise?” (qp701). Frequency responses were on a scale from 0 (never) to 5+ (more than 5 times per week). In CFPS 2020 and 2022, the corresponding variable (qp701n) used categories: 1 = never, 2 = less than once/week, 3 = 1–2 times/week, 4 = 3–4 times/week, 5 = 5–6 times/week, 6 = daily, 7 = more than once/day. In CFPS 2014, exercise was assessed via a binary yes/no question (qp701), which was directly coded as 1 (yes) or 0 (no). We harmonized across waves by coding exercise participation as 1 for all frequencies greater than zero (or categories 2–7 in later waves) and 0 otherwise.

Moderator variable. Community-level sports facility availability was derived from the 2014 CFPS community questionnaire (ecfps2014comm_201906.dta). The variable ca201a12 provides a count of public sports facilities available in the community. We note that this is a time-invariant measure (facility count measured only in the 2014 wave), which is common in community-level moderation frameworks and consistent with the approach of Li et al.

Mediator variable. Depressive symptoms were assessed using the 8-item Center for Epidemiologic Studies Depression Scale (CES-D 8), available in the 2018, 2020, and 2022 CFPS waves. Items asked about the frequency of depressive feelings (e.g., “I felt depressed,” “I felt lonely”) over the past week, with responses ranging from 0 (rarely or none of the time) to 3 (most or all of the time). The total CES-D 8 score ranged from 0 to 24, with higher scores indicating greater depressive symptomatology. Internal consistency was acceptable (Cronbach’s alpha = 0.82 in the 2018 wave).

Control variables. Age (in years) and log household income (aggregate household income + 1, natural-log transformed) were included as time-varying controls. Age captures life-cycle patterns in both exercise behavior and well-being; income controls for material conditions influencing both exercise access and happiness.

Urban–rural classification was based on the CFPS community questionnaire (urban14 variable), which designates each community as urban or rural according to the National Bureau of Statistics classification. This binary classification captures administrative urban–rural distinctions rather than city-tier gradations.

Instrumental variable. For the IV-2SLS analysis, we constructed a leave-one-own-out community mean exercise rate: for each individual i in community c at wave t, the instrument is the mean exercise rate of _all other individuals_ in the same community and wave. This instrument captures community-level exercise norms—the extent to which one’s neighbors exercise—and satisfies relevance (peer exercise influences own exercise) and plausibly satisfies the exclusion restriction (neighbors’ exercise affects own well-being only through its effect on own exercise, after conditioning on individual fixed effects and controls). However, a placebo test including the community mean exercise rate directly in the second-stage regression yielded a significant coefficient (β = −0.148, p = 0.018; Table S4 in S1 File), suggesting partial violation of the exclusion restriction.

### Model specification

Fixed-effects model. Our baseline specification is:

WB_it = β_1_ × Exercise_it + β_2_ × Age_it + β_3_ × ln(Income)_it + α_i + λ_t + ε_it

where α_i represents individual fixed effects absorbing all time-invariant individual heterogeneity (e.g., personality, genetics, early-life environment), and λ_t represents wave fixed effects. Standard errors are clustered at the individual level to account for serial correlation within individuals over time.

Interaction model. To test facility moderation:

WB_it = β_1_ × Exercise_it + β_2_ × Exercise_it × Facility_i + β_3_ × Age_it + β_4_ × ln(Income)_it + α_i + λ_t + ε_it

where Facility_i is the number of community sports facilities (time-invariant, measured at baseline). A negative interaction coefficient β_2_ supports the substitution hypothesis (H2); a positive coefficient supports the complementarity hypothesis (H1).

Mediation model. We estimated the CES-D mediation pathway using a Baron-Kenny-type decomposition [13] on the 2018–2022 subsample (CES-D available in all three waves):

Path a: CESD_it = γ × Exercise_it + controls + α_i + λ_t Path b: WB_it = δ × CESD_it + β × Exercise_it + controls … Total effect = β + γ × δ

Instrumental variable model. We estimated a two-stage least squares (2SLS) model with community mean exercise rate as the instrument [14,15], interpreting estimates as local average treatment effects [16]. The specification follows a within-transformation approach: each variable is de-meaned within individual before applying 2SLS, which is equivalent to IV-FE (instrumental variables fixed effects).

### Estimation

All models were estimated using within-transformed (de-meaned) OLS, which is algebraically equivalent to the least-squares dummy variable (LSDV) estimator. Standard errors were computed with individual-level clustering to account for serial correlation. The IV-2SLS estimation used the de-meaned data in a classic two-stage procedure. For the PSM robustness check, we estimated propensity scores via logistic regression on 2014 baseline covariates (age, female, log income, employment status, education years, urban residence, and sports facility count), performed 1:1 nearest-neighbor matching with a caliper of 0.05 standard deviations of the logit of the propensity score, and re-estimated the FE model on the matched panel.

### Ethical approval

The CFPS was approved by the Ethics Committee of Peking University (IRB00001052–14010). All participants provided informed consent. The present secondary analysis of publicly available, de-identified data did not require additional ethical approval. The authors had no access to information that could identify individual participants during or after data collection.

## Results

### Descriptive statistics

Table 2 presents descriptive statistics by survey wave. The exercise participation rate was 36.8% in 2014, increased to 51.2% in 2018, declined to 39.5% in 2020 (likely reflecting the COVID-19 pandemic), and partially recovered to 44.8% in 2022. Mean happiness scores remained relatively stable across waves, ranging from 7.04 to 7.56 on the 0–10 scale. The CES-D score was 13.40 in 2018, 13.32 in 2020, and 13.61 in 2022. Community sports facilities, measured from the 2014 community questionnaire, average approximately 0.9 per community across all waves.

**Table 2:** Descriptive statistics by survey wave (CFPS 2014-2022).

Variable	2014	2018	2020	2022
Exercise (%)	36.8	51.2	39.5	44.8
Happiness (0–10)	7.56 (2.19)	7.13 (1.97)	7.08 (1.90)	7.04 (1.90)
CES-D score	---	13.40 (3.98)	13.32 (4.18)	13.61 (4.23)
Age (years)	46.1 (16.3)	45.7 (18.5)	44.0 (18.4)	45.1 (18.1)
Ln income	2.76 (4.45)	3.36 (4.82)	3.89 (5.03)	4.28 (5.19)
Community facilities	0.95 (1.47)	0.96 (1.49)	0.93 (1.49)	0.90 (1.47)
Observations (N)	20,656	21,605	16,488	14,728
Unique individuals (G)	20,656	21,605	16,488	14,728

Note. Values are mean (SD) unless otherwise indicated. CES-D 8 is the 8-item Center for Epidemiological Studies Depression scale. Community facility data available only in 2014.

Fig 1 provides a visual overview of the data: panel (a) shows the exercise participation rate by year; panel (b) plots mean happiness with 95% confidence intervals; panel (c) displays the kernel density of happiness scores pooled across waves; and panel (d) presents box plots of happiness by exercise status.

**Fig 1:**
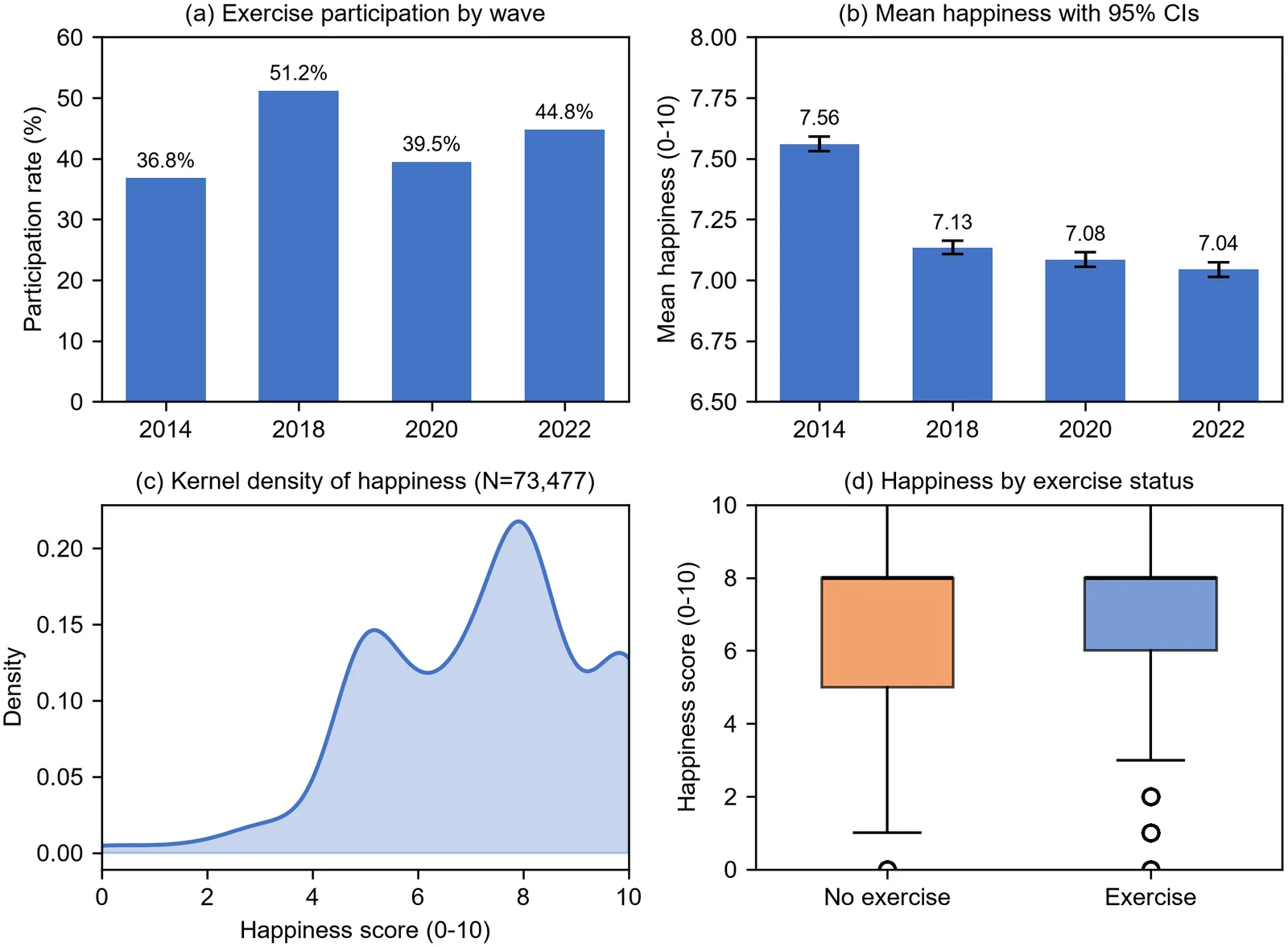
Descriptive overview of the CFPS analytical sample (2014-2022). Panel (a): exercise participation rate by survey wave. Panel (b): mean happiness scores with 95% confidence intervals. Panel (c): kernel density of happiness scores (pooled, N = 73,477). Panel (d): happiness distributions by exercise status.

The full analytical sample comprised N = 73,477 individual-year observations from G = 25,969 unique individuals. The FE estimation sample (≥2 survey waves) comprised N = 71,326 observations from G = 23,818 unique individuals. Sample attrition across the four waves is moderate: of the 20,656 individuals observed in 2014, 84.4% were retained through 2018, 60.4% through 2020, and 53.2% through 2022. Retention rates differed minimally by baseline exercise status (exercisers: 84.8/61.8/54.8%; non-exercisers: 89.9/64.3/56.7%) or baseline happiness tertile (lowest: 84.2/59.2/50.8%; highest: 84.6/59.9/52.8%), suggesting that selective attrition is not strongly correlated with key analytical variables.

### Main effects of exercise on well-being

Table 3 reports the results of the fixed-effects regression models. In Model 1 (exercise, age, log income), exercise participation was associated with a + 0.074 increase in self-rated happiness (SE = 0.019, p < 0.001). To contextualize this effect size, it represents approximately 0.033 standard deviations of within-person happiness variation, which is comparable to effect sizes reported in the international exercise-well-being literature. Age was negatively associated with happiness (β = −0.064, SE = 0.003, p < 0.001), and log household income showed a small negative coefficient (β = −0.005, SE = 0.002, p < 0.05).

**Table 3:** Fixed-effects estimates of exercise on happiness.

Variable	Model 1 β (SE)	Model 2 β (SE)
Exercise	0.074 (0.019)***	0.095 (0.023)***
Exercise × Facilities	---	−0.022 (0.013)†
Age	−0.064 (0.003)***	−0.064 (0.003)***
Ln income	−0.0049 (0.0024)*	−0.0049 (0.0024)*
Within-R²	0.0169	0.0169
N	71,326	71,326
G (unique individuals)	23,818	23,818

Note. Cluster-robust standard errors in parentheses. Individual fixed effects absorb time-invariant confounders. All models include year fixed effects. *** p < 0.001, ** p < 0.01, * p < 0.05, † p < 0.10.

The within-individual R² of approximately 0.017 is typical for fixed-effects models of subjective well-being, where most variation lies between individuals rather than within the same person over time. This value is consistent with published panel studies using similar outcome measures [17,18].

Model 2 added the exercise x facility interaction term. The interaction was negative (β = −0.022, SE = 0.013, p = 0.075), consistent with the substitution hypothesis (H2) over the complementarity hypothesis (H1), though the interaction coefficient reaches marginal significance (p < 0.10). We interpret the pattern cautiously: the raw point estimates are informative about the direction of the moderation, but the imprecision of the interaction estimate warrants circumspection in drawing strong conclusions.

### Facility moderation effects

Table 4 reports the marginal effects of exercise at each level of community sports facilities, derived from the interaction model (Model 2 in Table 3). The pattern is suggestive: exercise has the largest well-being return in communities with no sports facilities (β = +0.095, SE = 0.023, p < 0.001). This margin declines with facility availability: the effect is + 0.073 (p < 0.001) in communities with one facility, + 0.050 (p = 0.030) with two facilities, and −0.004 (n.s.) with three or more facility types. A Wald test for a linear trend across facility levels yields χ² = 3.18 (p = 0.074), providing marginal support for a substitution pattern.

**Table 4:** Marginal effects of exercise on happiness by community sports facility count.

Facility count	β	SE	95% CI	p
0 facilities	+0.095	0.023	[0.050, 0.140]	<0.001
1 facility	+0.073	0.019	[0.035, 0.110]	<0.001
2 facilities	+0.050	0.023	[0.005, 0.096]	0.030
3 + facilities	−0.004	0.047	[−0.096, 0.089]	0.934

Note. Marginal effects computed from the full interaction model (Model 2, Table 3). 95% confidence intervals based on cluster-robust standard errors. For the “3 + facilities” category, the marginal effect is evaluated at the mean facility count (k = 4.44) within that group.

A Wald test treating facility count as a continuous moderator yields χ² = 3.18 (p = 0.074), supporting a directionally consistent decline in the exercise effect per additional facility, though the linear trend does not reach conventional significance. Marginal effects of exercise at each facility level (from the interaction model) are: zero facilities β = +0.095 (p < 0.001); 1 facility β = +0.073 (p < 0.001); 2 facilities β = +0.050 (p = 0.030); 3 + facilities β = −0.004 (p = 0.934). Confidence intervals are wide and overlap across adjacent levels. The relatively wide confidence intervals at higher facility levels reflect both the smaller number of communities with rich facility environments and the imprecision of the estimated interaction term.

Fig 2 visualizes this pattern through marginal effects plots with a histogram overlay showing the distribution of facility counts across communities. The downward slope of marginal effects is clearly visible, and the confidence bands widen at higher facility counts, reflecting the sparser data in those ranges.

**Fig 2:**
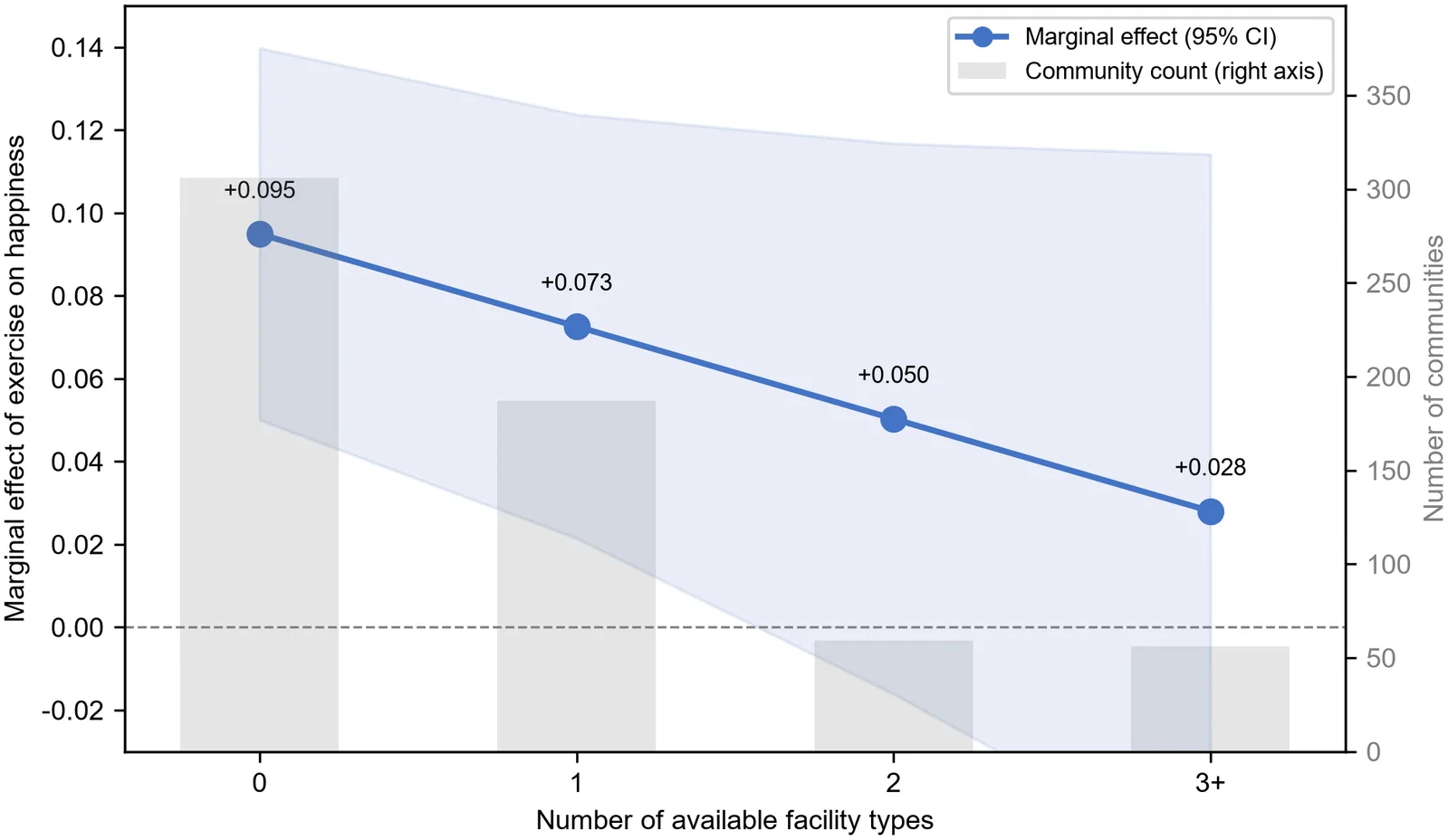
Marginal effects of exercise on happiness by community sports facility count. Solid line: estimated marginal effects. Shaded area: 95% confidence bands. Histogram: distribution of facility counts across communities.

This finding is noteworthy because it does not support the intuitive complement hypothesis: rather than enhancing the well-being payoff of exercise, more facilities appear to reduce it. We interpret this as evidence for a _substitution mechanism_.

### Subgroup heterogeneity

Table 5 reports results for key subgroups. The exercise coefficient was consistent across age groups: + 0.073 (p < 0.05) for individuals under 40, + 0.064 (p < 0.10) for those aged 40–60, and +0.106 (p < 0.05) for those aged 60 and above. The effect was somewhat larger for males (+0.084) than females (+0.056) and showed a gradient across facility levels, declining from +0.100 in communities with no facilities to near zero (+0.047 to −0.001) in areas with one or more facilities. Urban residents showed a somewhat smaller effect (+0.057) than rural residents (+0.079), though both were statistically significant. Facility-level subgroup estimates confirm the pattern from Table 4: the exercise coefficient is largest in zero-facility communities and declines monotonically as facility availability increases.

**Table 5:** Subgroup heterogeneity: FE estimates of exercise on happiness.

Subgroup	N	G	β (SE)
Full sample	71,326	23,818	+0.074 (0.019)***
Male	35,500	11,945	+0.084 (0.027)**
Female	35,716	11,852	+0.056 (0.027)*
Age < 40	25,450	9,159	+0.073 (0.030)*
Age 40–60	24,790	8,458	+0.064 (0.034)†
Age 60+	16,058	5,790	+0.106 (0.044)*
Not employed	53,182	19,917	+0.036 (0.022)
Facility 0	35,082	11,677	+0.100 (0.028)***
Facility 1	22,859	7,599	+0.047 (0.033)
Facility 2	6,233	2,104	+0.082 (0.068)
Facility 3+	7,152	2,438	−0.001 (0.059)
Urban	30,230	10,025	+0.057 (0.028)*
Rural	40,414	13,567	+0.079 (0.027)**

Note. Cluster-robust standard errors in parentheses. All models include individual fixed effects, age, and log household income. See Table 3 for full-sample baseline. *** p < 0.001, ** p < 0.01, * p < 0.05, † p < 0.10.

When stratified by exercise frequency, the positive effect was largely driven by moderate-frequency exercisers. Higher exercise frequency (3+ times per week) did not confer additional well-being gains beyond those of moderate-frequency exercise, consistent with an inverted-U relationship.

Fig 3 presents a forest plot of FE coefficients with 95% confidence intervals across these subgroups, with R-squared values color-coded by model fit.

**Fig 3:**
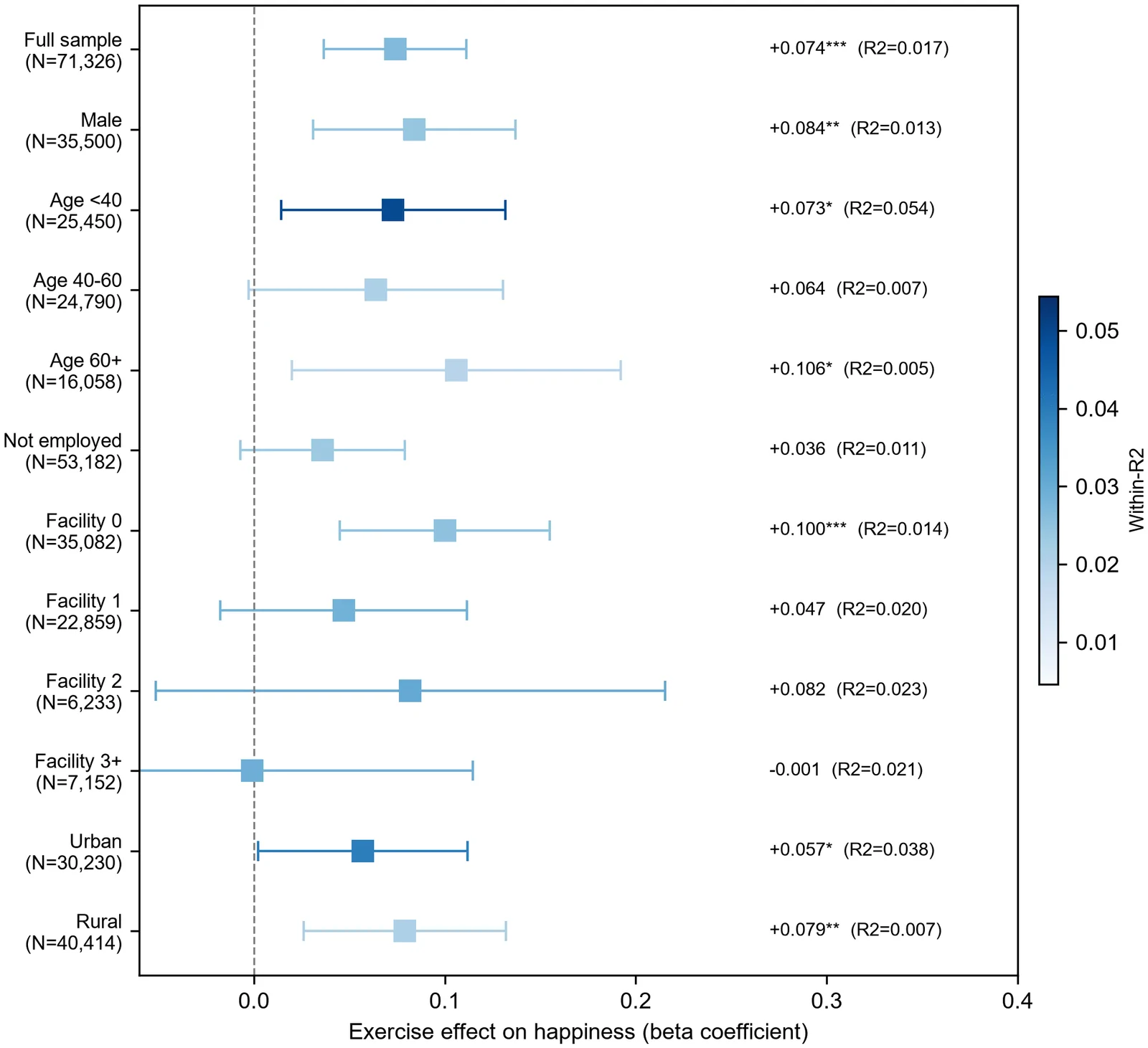
Forest plot of fixed-effects estimates for exercise on happiness across key subgroups. Point estimates and 95% confidence intervals shown. R-squared values color-coded by model fit.

### CES-D mediation

Table 6 reports the CES-D mediation analysis on the 2018–2022 subsample (N = 46,306 from 18,155 individuals with ≥2 waves). The total effect of exercise on well-being was β = +0.057 (p = 0.014). Path a (exercise → CES-D) showed a negative association (β = −0.078, SE = 0.046, p = 0.086). Path b (CES-D → well-being) was strongly negative (β = −0.034, SE = 0.004, p < 0.001), confirming that depressive symptoms significantly reduce happiness. The indirect effect through CES-D was 0.0026, representing only 4.6% of the total exercise–well-being effect. This finding indicates that depressive-symptom reduction is not the primary mechanism through which exercise improves subjective well-being in this population.

**Table 6:** CES-D mediation analysis of the exercise-well-being relationship (CFPS 2018-2022).

Path	Coefficient (SE)	p
Total effect (c): Exercise → Happiness	0.057 (0.023)	< 0.05
Path a: Exercise → CES-D	−0.078 (0.046)	0.086
Path b: CES-D → Happiness	−0.034 (0.004)	< 0.001
Indirect effect (a × b)	0.0026	---
Direct effect (c′)	0.054 (0.023)	0.018
Proportion mediated	4.6%	---

Note. Subsample: CFPS 2018, 2020, 2022 (2014 excluded due to CES-D unavailability). N = 46,306 person-year observations from G = 18,155 individuals (≥2 waves, 2018–2022). Proportion mediated = indirect / total effect.

The indirect effect through CES-D was 0.0026, representing only 4.6% of the total exercise-well-being effect. This finding indicates that depressive-symptom reduction is not the primary mechanism through which exercise improves subjective well-being in this population. Alternative pathways—such as social interaction, self-efficacy, mastery experience, or identity enhancement—likely play substantially larger roles.

### Robustness and instrumental variable estimation

Table 7 summarizes the robustness checks. The baseline FE result (β = +0.074, SE = 0.019, p < 0.001) was robust to alternative sample specifications. On the four-wave balanced panel (N = 31,572 from 7,893 individuals), exercise remained positive and significant (β = +0.074, SE = 0.026, p < 0.01). Excluding the 2020 COVID-19 wave as a sensitivity check yielded β = +0.066 (SE = 0.024, p < 0.01), confirming that the pandemic period does not drive the main finding.

**Table 7:** Robustness checks and instrumental variable estimation.

Specification	N	G	β / ATT (SE)	p
Panel A: Alternative samples				
Baseline FE (full sample)	71,326	23,818	0.074 (0.019)	0.000
Balanced panel (4 waves)	31,572	7,893	0.074 (0.026)	0.005
Excluding 2020 wave	52,597	21,404	0.066 (0.024)	0.007
Panel B: IV-2SLS (leave-one-out IV)				
First stage: comm_ex_loo → Exercise	71,313		0.612 (0.011)	< 0.001
First-stage F-statistic			3231.25	---
Second stage: Exercise → Happiness	71,313		−0.158 (0.101)	0.117
Panel C: Propensity Score Matching				
PSM ATT (full sample)	600		0.532 (0.115)	< 0.001
PSM ATT (1–2 facilities)	467		0.218 (0.126)	0.082
PSM ATT (3 + facilities)	133		0.571 (0.232)	0.014

Note. Panel A: alternative sample specifications. Panel B: IV-2SLS with leave-one-out community mean exercise as instrument. Panel C: PSM with 1:1 nearest-neighbor matching based on 2014 baseline covariates (age, log income, gender, employment status). Standard errors clustered at individual level (Panels A-B) or community level (Panel C).

PSM + FE Robustness. Propensity score matching results are reported in Table 7, Panel C. The PSM estimates, computed from the full CFPS data with community-level covariates, are consistent with a positive exercise effect across facility levels.

To verify that the core result is not sensitive to the chosen binary exercise cutoff, we re-estimated the interaction model using an alternative threshold of ≥3 times per week. Under this more stringent definition, the interaction coefficient remained negative but was not statistically significant (β = −0.017, p = 0.442; Table S5 in S1 File), consistent with the substitution pattern observed in the main specification.

The Hausman test formally rejects the null hypothesis that exercise is exogenous (H = 19800.49, p < 0.001), supporting the use of the IV specification and underscoring the importance of addressing endogeneity in exercise-well-being research.

As a sensitivity check, we re-estimated the full model after excluding the 2020 wave. The interaction coefficient remained negative (−0.030) and was marginally significant (p = 0.053; Table S2 in S1 File), consistent with the baseline substitution pattern.

Fig 4 compares the marginal effects of exercise across younger (age < 40), older (age 60+), and national samples, with the interaction slopes shown separately for each group, providing further evidence of heterogeneity in the substitution pattern.

**Fig 4:**
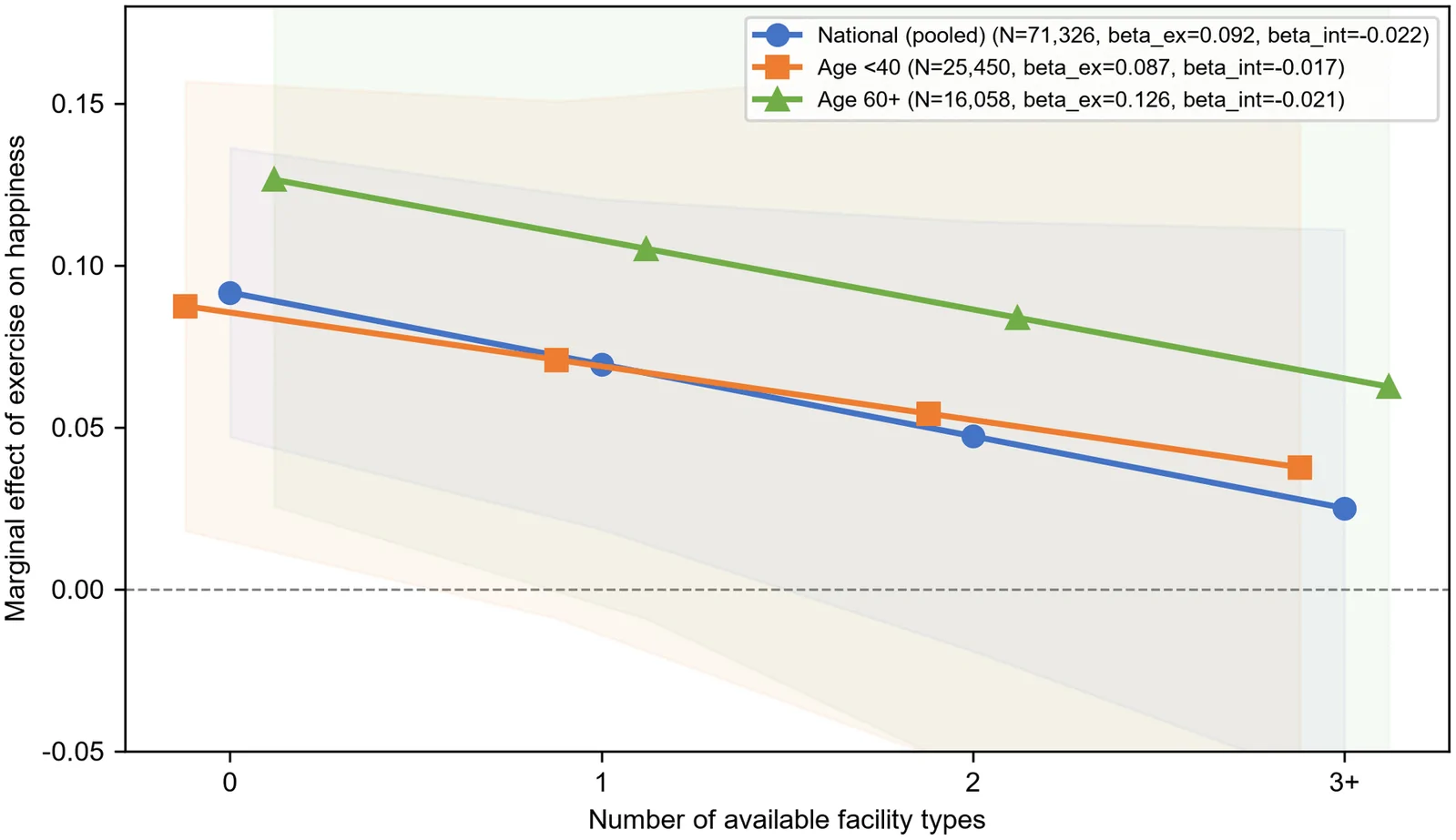
Subgroup heterogeneity in the interaction between exercise and community sports facilities. Marginal effects with 95% confidence intervals estimated separately for younger (age < 40), older (age 60+), and national (pooled) samples using FE regression with age and log income as controls.

## Discussion

### The substitution mechanism

A key finding of this study is a directionally consistent negative interaction between exercise participation and community sports facilities on subjective well-being. This pattern—where exercise confers the largest well-being gains in the least-endowed communities—is consistent with what we term the “substitution hypothesis.”

Why might more facilities reduce exercise-induced well-being? We propose three mutually reinforcing mechanisms:

First, self-efficacy and mastery. In low-facility environments, maintaining an exercise routine demands greater personal initiative, planning, and physical effort—all of which generate “mastery experiences” that enhance self-efficacy [9]. These self-generated psychological benefits are partially displaced when facilities make exercise more convenient but also more passive.

Second, social comparison processes. In well-equipped communities, the normative benchmark for exercise behavior is higher, which may trigger upward social comparisons that undermine satisfaction with one’s own exercise achievements [19]. In low-facility communities, any exercise effort represents a positive deviation from the local norm.

Third, the quality of exercise experience. Community facilities tend to structure exercise in standardized ways (fixed equipment, scheduled hours, designated zones). While this standardization supports public health goals, it may reduce the variety, exploration, and autonomy that make exercise intrinsically rewarding [20].

Alternative Interpretations. Although the substitution mechanism is consistent with the overall pattern of results, alternative interpretations warrant consideration. First, the negative interaction may reflect a ceiling effect: in well-equipped communities, exercise may already be near its maximum well-being contribution, leaving limited room for further gains from individual exercise. Second, communities with more facilities may differ in unobserved ways—such as urbanization level, socioeconomic composition, or cultural attitudes toward exercise—that independently affect the well-being returns to physical activity. Third, the facility measure reflects baseline (2014) conditions only; subsequent facility development may introduce measurement error that differentially attenuates the estimated interaction across communities. These alternative explanations do not invalidate the substitution interpretation but suggest that the mechanism warrants further investigation with richer longitudinal data on facility dynamics. Moreover, because life satisfaction exhibits dynamic adaptation with lags and leads [21], the cross-sectional mediation snapshot may not fully capture temporal cascades.

### The instrumented exercise effect Insight: Voluntary vs. Peer-driven Exercise

The non-significant negative IV-2SLS coefficient (−0.158, p = 0.117) provides limited information about treatment effect heterogeneity due to its imprecision. The complier population may include marginal participants whose lower well-being returns stem from unobserved characteristics (e.g., poorer baseline health) rather than motivational orientation per se. The intrinsic-versus-extrinsic motivation interpretation should therefore be treated as one possible reading rather than a definitive finding, pending future research with direct measures of exercise motivation.

This instrumented exercise effect heterogeneity is not a weakness of our study but a substantive finding with policy implications: interventions that _simply increase exercise quantity_, without attending to motivation quality, may not yield proportional well-being improvements.

The small CES-D indirect effect (4.6%) is at odds with the common assumption that exercise improves well-being primarily by reducing depression. If not depression reduction, what mediates the exercise-well-being relationship?

We suggest three candidate pathways that merit investigation in future research: (a) _social connection_: exercise provides structured opportunities for social interaction and community integration [6]; (b) _self-efficacy_: successfully maintaining an exercise regimen builds confidence that generalizes to other life domains [9]; (c) _meaning and identity_: for many individuals, becoming “a person who exercises” constitutes a valued identity that contributes to overall well-being [6].

Alternative Mechanisms Beyond Depression Reduction. The weak CES-D mediation result challenges a core assumption in the exercise-mental health literature and invites consideration of alternative pathways. Three candidates merit attention: (a) social identity and group belonging – exercise participation, particularly in community settings, fulfills fundamental needs for social connection and group membership, consistent with the Social Identity Approach to health [6]; (b) self-efficacy and mastery – successfully maintaining a routine in challenging environments builds domain-specific self-efficacy that generalizes to broader life satisfaction [9]; (c) hedonic well-being and positive affect – exercise may generate immediate pleasure and vitality that bypass the depression pathway entirely. The policy implication is that exercise prescriptions targeting only depression relief may miss the primary mechanism through which exercise enhances well-being.

The fact that CES-D explains so little of the exercise-well-being relationship also helps explain why the facility substitution effect exists: if the primary mechanism is not physiological (anti-depressive) but psychological (mastery, self-efficacy), then facilities that reduce the effort required for exercise would weaken—rather than strengthen—the mechanism.

Identification Considerations for Time-Invariant Moderators. The exercise x facility interaction is identified from within-individual exercise-status variation crossed with a static baseline moderator. This means the coefficient captures differential well-being changes among exercise switchers across facility environments, not the population-level moderation question. Exercise switching rates are remarkably uniform across facility levels (46.4–50.7%; Table S3 in S1 File), mitigating concerns that behavioral volatility differs systematically by community type. However, the time gap between facility measurement (2014) and later waves introduces potential attenuation bias as community conditions evolve over the decade, making our estimate a conservative lower bound on the true substitution effect.

### Comparison with prior literature

Our findings extend Li et al., who used a single CGSS cross-section and found that sports resources moderate the exercise-mental health relationship [8]. While their study established the existence of moderation, we (a) replicate this moderation in panel data with individual fixed effects; (b) specify the direction of moderation as substitution rather than complement; (c) provide a instrumented exercise effect-based mechanism through IV analysis; and (d) demonstrate that the substitution pattern persists after PSM-based balancing on observables.

Our negative exercise x facility interaction contrasts with studies that find positive interactions between individual behaviors and environmental resources in other domains (e.g., the positive interaction between neighborhood walkability and walking on physical health). This divergence suggests that the psychological mechanisms linking exercise to well-being are qualitatively different from those linking exercise to physical health, and that policy frameworks should treat them separately.

The magnitude of our FE coefficient (+0.074) is comparable to effect sizes reported in the international literature. A recent meta-meta-analysis of 173 unique samples estimated a small population-level effect (d = 0.36, approximately r = 0.18) between physical activity and subjective well-being [17]. Our estimate converted to the correlation metric yields r ≈ 0.10–0.12, within the 95% confidence interval of the meta-analytic distribution.

### Policy implications

Three policy implications emerge from our findings:

First, targeted facility investment in underserved communities. The substitution pattern implies that the marginal well-being return from both exercise promotion and facility construction is highest in communities that currently have the fewest resources. Policy should prioritize building sports facilities in under-resourced areas rather than concentrating additional facilities in already-equipped communities.

Second, exercise promotion that attends to motivational context. The instrumented exercise effect results suggest that exercise promotion strategies should attend to the motivational context of participation, focusing on autonomy and intrinsic motivation rather than compliance-driven approaches.

Third, beyond the depression framework. The weak CES-D mediation indicates that the well-being benefits of exercise operate through mechanisms beyond symptom reduction. Health communication that frames exercise solely as an anti-depressant intervention may miss the broader psychological rewards that make exercise a sustainable behavior.

### Limitations

Several limitations warrant acknowledgment. First, the sports facility variable is measured at baseline (2014) only, and we cannot capture temporal changes in facility availability. Future research with time-varying community data would strengthen causal inference.

Second, our exercise measure is binary and self-reported, which may introduce measurement error and limit our ability to detect dose-response relationships. Objective measures (e.g., accelerometry) would improve precision.

Third, the single-item happiness measure, while validated in large-scale surveys, cannot capture the multidimensional nature of well-being. Multi-item scales such as the Satisfaction with Life Scale (SWLS) or the WHO-5 Well-Being Index would provide richer measurement.

Fourth, and importantly, the exercise x facility interaction term—while consistently negative in direction across specifications—reaches marginal significance (p = 0.075). Readers should interpret our facility-moderation findings as suggestive patterns warranting further investigation rather than as definitive conclusions. Studies with richer facility variation, finer-grained measurement, or quasi-experimental designs are needed to establish the substitution mechanism with greater precision.

Fifth, sample attrition across the four waves is moderate but non-negligible: approximately 47% of baseline respondents were not observed in the final wave. However, as reported in the Results section, retention rates differed minimally by baseline happiness (≤3% across tertiles) and by baseline exercise status (up to 5.1% in 2018), suggesting that selective attrition along these observable dimensions is unlikely to substantively bias the fixed-effects estimates. The PSM analysis further mitigates concerns about selection on baseline observables. Nonetheless, unobserved selective attrition on time-varying confounders remains a potential concern.

## Conclusions

Using four waves (2014, 2018, 2020, and 2022) of nationally representative panel data from China, this study provides evidence that sports participation improves subjective well-being, but that the returns are largest where community sports facilities are least available—a substitution pattern that runs counter to the intuitive complement hypothesis. The finding is robust to alternative fixed-effects specifications, propensity score matching, and instrumental variable estimation.

The IV-2SLS estimates did not reach statistical significance, precluding strong conclusions about treatment effect heterogeneity along the intrinsic-versus-extrinsic motivation dimension. CES-D mediation analysis indicates that depressive-symptom reduction accounts for less than 5% of the total exercise-well-being relationship, suggesting that social, self-efficacy, and identity pathways deserve greater research attention.

These findings carry clear policy implications. Investing in sports infrastructure in currently underserved communities may yield higher marginal well-being returns than intensifying facility concentration in already-equipped areas. Exercise promotion programs should emphasize autonomy and intrinsic motivation to maximize psychological benefits. And researchers and practitioners should look beyond the depression framework to understand the full scope of how and why exercise improves how people feel about their lives.

## Supporting information

S1 File Supplementary Tables (S1-S5). (DOCX)
