# Supplementary material for: Sports facilities as substitutes for exercise-induced well-being: Evidence from four-wave CFPS panel data
Source: PLoS One. 2026 Sep 30;21(9):e0359511. doi: 10.1371/journal.pone.0359511 (PMC13626477; doi:10.1371/journal.pone.0359511)
Supplement: Supplementary file 1 [file pone.0359511.s001.docx]

**Supplementary Tables**

**Table S1. Cross-sectional OLS estimates by survey wave (no individual fixed effects).**

| **Wave** | **N** | **Exercise β** | **Exercise × Facility β** | **Interaction p** |
| --- | --- | --- | --- | --- |
| 2014 | 20,656 | +0.468 | +0.021 | 0.288 |
| 2018 | 21,605 | +0.280 | -0.002 | 0.932 |
| 2020 | 16,488 | +0.152 | +0.023 | 0.244 |
| 2022 | 14,728 | +0.261 | -0.002 | 0.933 |

*Note. Cross-sectional OLS estimates (no individual fixed effects) with community-level sports facility count as moderator. Standard errors clustered by community. Controls include age and log household income. The interaction term is not statistically significant in any wave, consistent with the directionally consistent but limited precision of the moderating effect observed in the FE models.*

**Table S2. Sensitivity analysis excluding the 2020 COVID-19 wave.**

| **Specification** | **Exercise β** | **Exercise × Facility β** | **Interaction p** | **N** | **G** |
| --- | --- | --- | --- | --- | --- |
| Full sample (4 waves) | +0.095 | -0.022 | 0.075 | 71,326 | 23,818 |
| Excluding 2020 wave | +0.094 | -0.030 | 0.053 | 52,597 | 21,404 |

*Note. Individual FE models with year FE, age, and log income. Excluding 2020 increases interaction magnitude and yields marginal significance (p = 0.053).*

**Table S3. Exercise switching rates by baseline community sports facility level.**

| **Facility Level** | **Individuals (G)** | **Exercise Switchers** | **Switching Rate (%)** |
| --- | --- | --- | --- |
| 0 facilities | 11,677 | 5,716 | 49.0 |
| 1 facility | 7,599 | 3,850 | 50.7 |
| 2 facilities | 2,104 | 977 | 46.4 |
| 3+ facilities | 2,438 | 1,179 | 48.4 |

*Note. Switchers = individuals who changed exercise status between any two waves. Rates are uniform across facility levels (46.4–50.7%), supporting FE identification validity.*

***Table S4. Placebo test for the exclusion restriction: community mean exercise rate in the second-stage regression.***

| **Variable** | **β (SE)** | **p** | **N** |
| --- | --- | --- | --- |
| Exercise (individual-level) | 0.084 (0.020) | 0.000 | 71,313 |
| Community leave-one-out mean exercise rate | -0.148 (0.063) | 0.018 | 71,313 |

***Table S5. Alternative exercise threshold (≥3 times/week): interaction model results.***

| **Variable** | **Model 1 β (SE)** | **Model 1 p** | **Model 2 β (SE)** | **Model 2 p** |
| --- | --- | --- | --- | --- |
| Exercise (≥3 times/week) | 0.064 (0.024) | 0.007 | 0.092 (0.056) | 0.100 |
| Exercise × Facilities | --- | --- | -0.017 (0.022) | 0.442 |
| Within-R² | 0.0106 | --- | 0.0132 | --- |
| N | 30,971 | --- | 13,520 | --- |
| G | 11,584 | --- | 5,054 | --- |

*Note. Individual FE models with year FE, age, and log income. Model 1: exercise main effect only. Model 2: exercise + facility interaction. The alternative threshold (≥3 times/week) yields a smaller subsample but confirms the directionally consistent negative interaction.*
